# Trousseau Syndrome Manifesting as Recurrent Embolic Stroke in ROS1-Positive Non-Small-Cell Lung Cancer: A Case Report

**DOI:** 10.3390/jcm15218743

**Published:** 2026-11-09

**Authors:** Tsung-Ta Wu, Shih-Lung Cheng

**Affiliations:** 1 Department of Internal Medicine, Far Eastern Memorial Hospital, New Taipei City 220216, Taiwan; 2 Department of Chemical Engineering and Materials Science, Yuan-Ze University, Taoyuan City 320315, Taiwan

**Keywords:** lung cancer, ROS1, Trousseau syndrome, embolic stroke

## Abstract

Background: Trousseau syndrome is a paraneoplastic hypercoagulable state manifesting as recurrent thromboembolism. Lung adenocarcinomas harboring specific driver mutations, such as ROS1 rearrangements, are associated with a significantly increased risk of these events and related complications. Case presentation: A 61-year-old non-smoking female with stage IV ROS1-positive lung adenocarcinoma developed bilateral cerebral and cerebellar infarctions. While aspirin therapy was initiated, she still experienced a recurrent ischemic stroke involving the left middle cerebral artery, leading to a diagnosis of Trousseau syndrome. However, the course was further complicated by pneumonia and respiratory failure. Anticoagulation with apixaban was initiated. A third embolic event subsequently occurred, involving a right middle cerebral artery occlusion with associated subarachnoid hemorrhage and hemorrhagic transformation. Despite intensive management, the patient’s condition deteriorated, and she passed away. Conclusions: Trousseau syndrome significantly impairs the prognosis of lung cancer patients, especially with specific driven genes, such as ROS-1 gene re-arrangement. This case highlights the deteriorating prognosis of cancer-associated thrombosis, which can be refractory and requires heightened clinical vigilance.

## 1. Introduction

Trousseau syndrome is a distinctive paraneoplastic syndrome in cancer patients. It is characterized by hypercoagulability, manifesting as recurrent or migratory thromboembolic events [1]. Lung cancer harboring certain mutated driver genes has been reported to be associated with a higher risk of thromboembolism [2]. Prevalence of ROS1 (Proto-oncogene tyrosine-protein kinase 1) rearrangement in non-small-cell lung cancer (NSCLC) is approximately 1–2%. Compared to patients with other genetic alterations (e.g., epidermal growth factor receptor (EGFR), Kirsten rat sarcoma (KRAS), ROS1 and anaplastic lymphoma kinase (ALK)-positive patients exhibit a significantly higher risk of thrombosis. While the vast majority of literature focuses on venous thromboembolism (VTE), the literature regarding arterial thromboembolism or recurrent ischemic stroke in these patients remains relatively scarce [3]. In this report, we described a case of Trousseau syndrome manifesting as recurrent embolic cerebral stroke in a patient with ROS1-positive non-small-cell lung cancer.

## 2. Case Presentation

**Figure 1:**
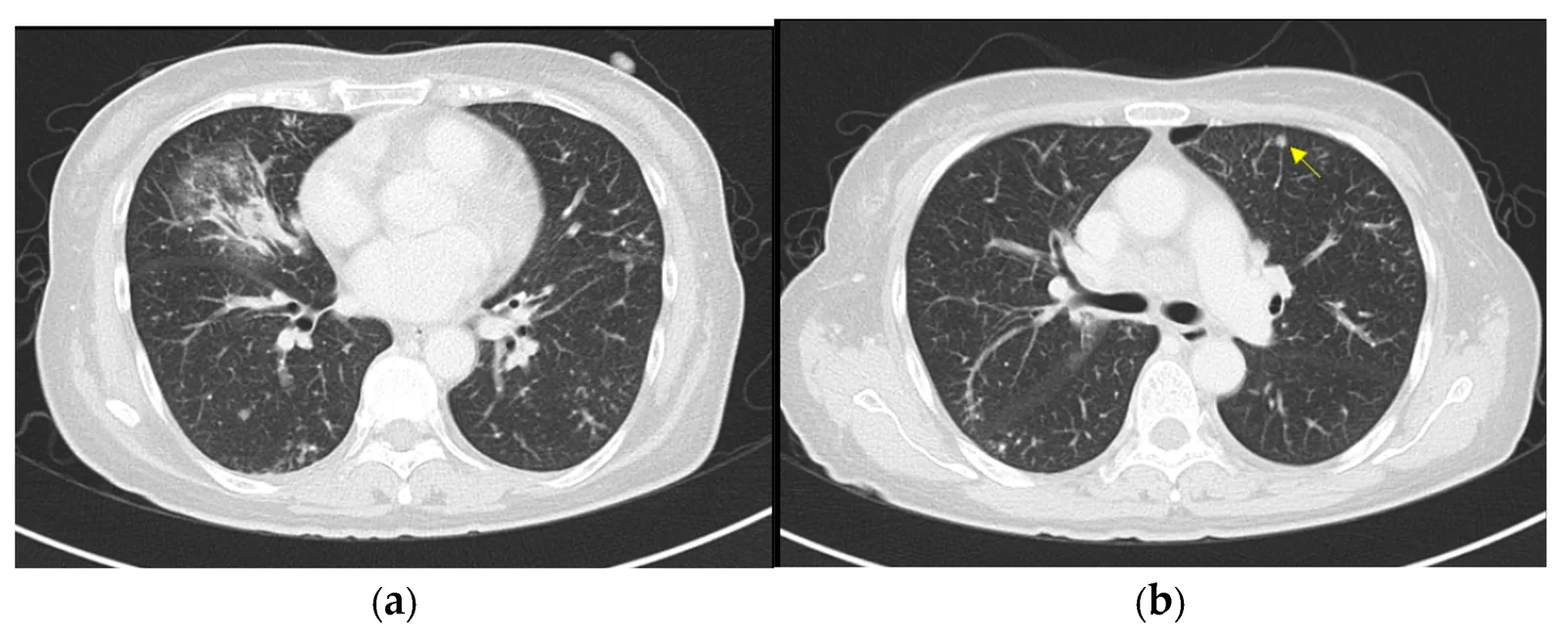
(**a**) Chest CT scan showing a consolidated patch (3.5 × 1.8 × 1.8 cm) in the right middle lobe, subsequently confirmed as lung adenocarcinoma via CT-guided biopsy. (**b**) Multiple metastatic nodules in the bilateral lungs. A nodule in the contralateral lung is indicated (arrow).

A 61-year-old non-smoking woman with no significant medical history presented with a productive cough and unintentional weight loss for one month. Stage IV lung adenocarcinoma (cT4N3M1c) was confirmed via computed tomography-guided biopsy of a right middle lobe tumor. Metastases involved bilateral lungs, mediastinal lymph nodes, vertebrae, and malignant pericardial effusion. (Figure 1) Brain magnetic resonance imaging (MRI) revealed no brain metastasis or intracranial lesions. Molecular testing, including EGFR, ALK, ROS1 and programmed death-ligand 1(PD-L1) expression, was performed to evaluate targeted therapy options.

**Figure 2:**
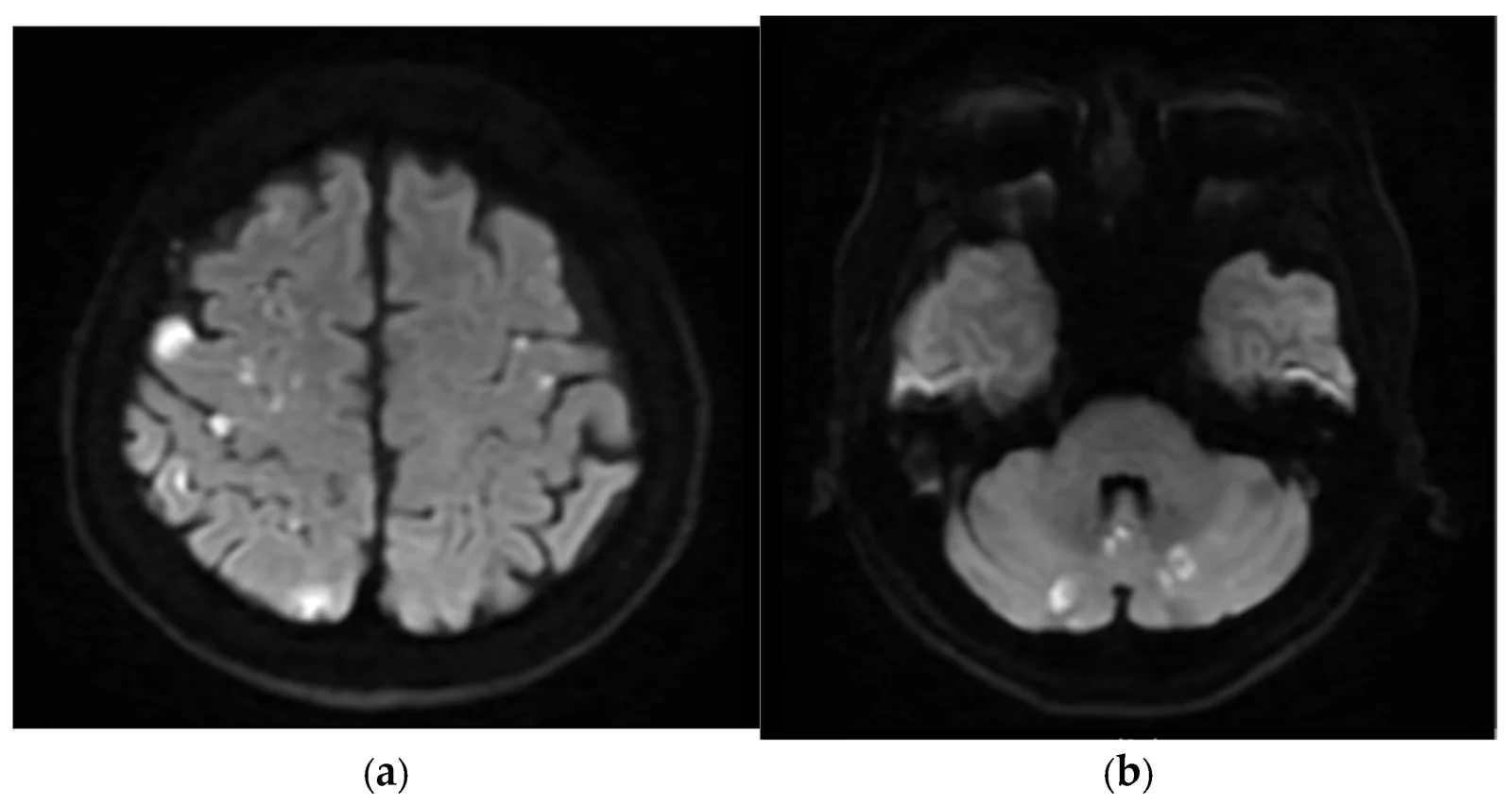
Diffusion-weighted brain MRI showing multiple embolic infarctions within the bilateral cerebrum (**a**) and bilateral cerebellum (**b**).

Before driver mutation results were available, the patient developed acute altered mental status over two days, including fluctuating disorientation, incoherent speech, and urinary incontinence. Neurological examinations showed isocoric pupils with normal light reflexes and no focal limb weakness. Brain CT revealed right frontoparietal hypodense lesions, while MRI confirmed acute bilateral cerebral and cerebellar embolic infarctions (Figure 2). Magnetic resonance angiography images showed no evidence of vascular disease, with the major intracranial arteries, bilateral proximal internal carotid arteries, and vertebral arteries all remaining patent. Thrombolytic therapy and mechanical thrombectomy were deferred due to the late presentation and absence of large vessel occlusion. The patient started on antiplatelet therapy with aspirin for ischemic stroke and was admitted to the pulmonary ward.

An etiological workup for ischemic stroke was performed. Workups to exclude cardioembolic stroke, including electrocardiogram, 24-h Holter monitor, and transthoracic echocardiography, were negative for cardiac rhythm abnormalities or left atrial thrombi. Transesophageal echocardiography was not performed, considering its invasive nature and the absence of thrombus on transthoracic echocardiography. Although prothrombin time (PT), international normalized ratio (INR), activated partial thromboplastin time (aPTT), and fibrinogen were normal, D-dimer (>35.20 mg/L FEU) and fibrin degradation products (FDP) (125.9 μg/mL) were significantly elevated. The patient has no history of other stroke risk factors, such as smoking or hypertension. Her lipid profile was within normal limits (LDL: 104 mg/dL, total cholesterol: 146 mg/dL, triglycerides: 81 mg/dL). Subsequently, ROS1 rearrangement was detected in 94% of cells via fluorescence in situ hybridization (FISH) assay. Crizotinib therapy was initiated, and the patient’s mental status remained stable.

**Figure 3:**
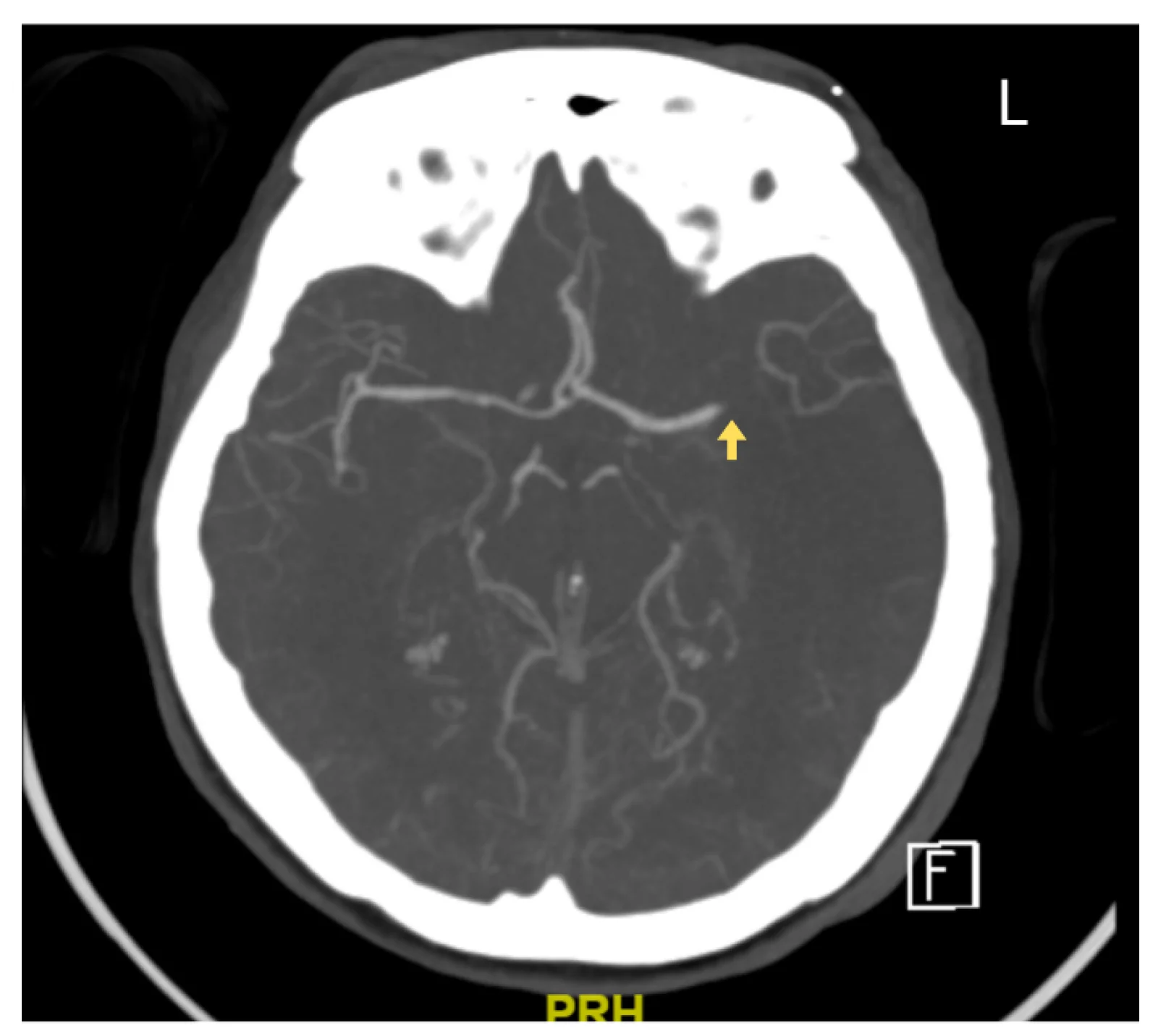
Brain CTA showing left M1 MCA occlusion as indicated by the arrow.

On admission day five, the patient experienced recurrent altered consciousness with agitation and inability to follow commands. Clinical status rapidly deteriorated due to new-onset pneumonia, resulting in respiratory failure that required emergent intubation and intensive care unit (ICU) transfer. The following day, right-sided hemiparesis developed (muscle strength 4 to 1), and computed tomography angiography (CTA) of brain confirmed a left M1-segment MCA occlusion (Figure 3). Thrombolytic therapy was contraindicated due to a recent stroke within a month. Meanwhile, mechanical thrombectomy was not performed considering the limited benefit, as indicated by an Alberta Stroke Program Early CT Score (ASPECTS) of 5 and a time window of more than 12 h beyond the last known well time.

Neurology and neurosurgery consultants suspected a hypercoagulable state consistent with Trousseau syndrome, leading to a switch from aspirin to apixaban since ICU Day 9. Apixaban was administered at a dose of 2.5 mg twice a day via a nasogastric tube without interruption. The dosage was chosen taking into account the patient’s old age, low body weight (49.7 kg), preserved renal function and concomitant crizotinib therapy, as well as the heightened risk of hemorrhagic transformation from her recent ischemic stroke. Renal and hepatic functions, as well as platelet counts, were closely monitored during anticoagulation therapy and remained within normal limits.

**Figure 4:**
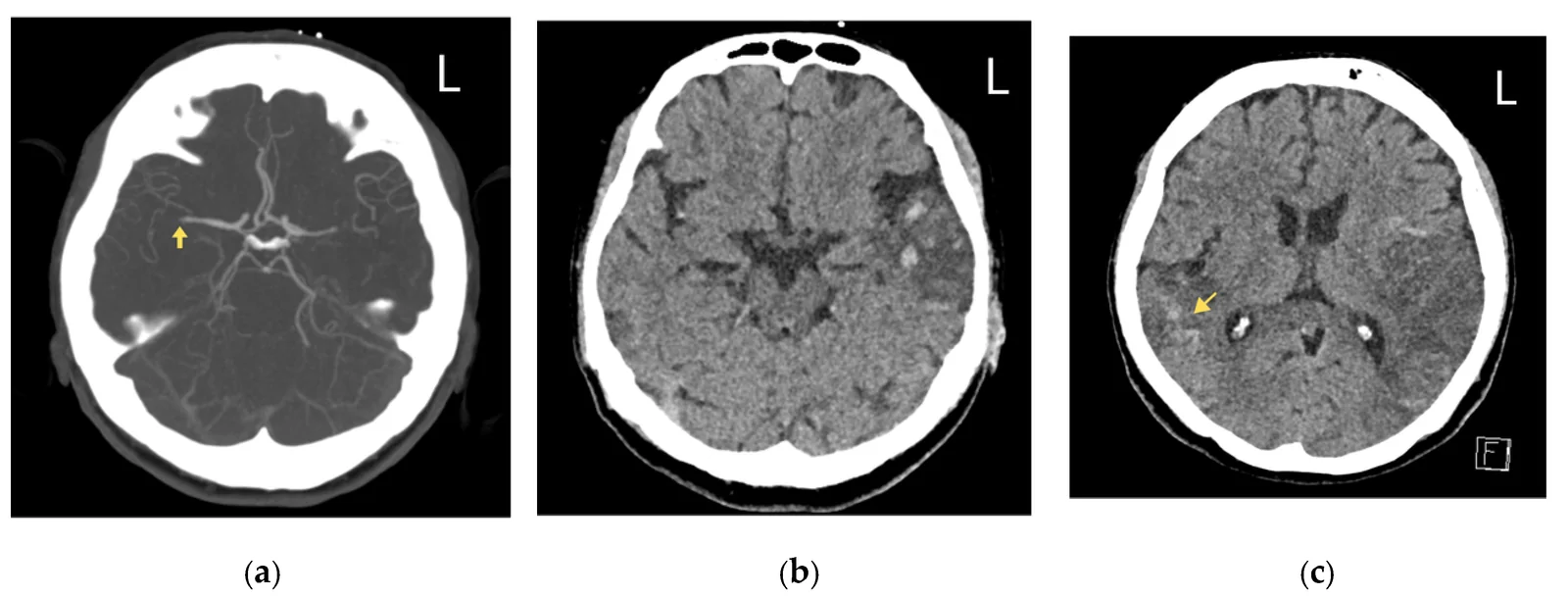
(**a**) Right distal M1 MCA occlusion on CTA as indicated by the arrow. (**b**) Hemorrhagic transformation within left MCA territory. (**c**) Acute SAH in the right parietal and temporal lobes as indicated by the arrow.

Despite anticoagulation, a recurrent stroke occurred on ICU Day 15, with left-sided muscle strength declining from 4 to 2. Repeat CTA revealed several findings, including a right distal M1 segment MCA occlusion, acute subarachnoid hemorrhage (SAH) in the right parietal and temporal lobes, and a large acute infarct with hemorrhagic transformation in the left frontal lobe and left MCA territory (Figure 4). Apixaban was subsequently withheld. No reversal agents were administered.

Once again, neither thrombolytic therapy nor mechanical thrombectomy was indicated. The patient’s condition remained critical, characterized by ventilator dependence, persistently impaired consciousness, and dilated pupils. On ICU day 22, she developed bradycardia and hypotension, followed by cardiac arrest. Cardiopulmonary resuscitation was performed for 30 min without return of spontaneous circulation, and the patient passed away. The detailed chronological sequence of clinical events, neuroimaging findings, and therapeutic interventions throughout the patient’s hospitalization is summarized in Table 1.

## 3. Discussion and Conclusions

Trousseau syndrome is a malignancy-associated hypercoagulability manifesting as recurrent or migratory thromboembolic events, including venous thromboembolism (VTE), arterial thrombosis, and disseminated intravascular coagulation (DIC). It is the second leading causes of death in cancer patients, after death from cancer itself [1]. Lung cancer has one of the highest incidences of thromboembolic events, occurring throughout the disease course [4]. The incidence rate of VTE in patients with lung cancer ranges from 1% to 20%. A meta-analysis reported pooled VTE incidence rates of 5% in patients with lung cancer, with higher rates observed in patients with advanced disease, adenocarcinoma histology, and those receiving chemotherapy or radiotherapy [5]. The 6-month cumulative incidence of arterial thromboembolic events (ATE) in patients with lung cancer is approximately 8.3% [6]. This risk is particularly pronounced shortly before and after cancer diagnosis, with the highest incidence occurring within the first year [7].

The incidence rate of Trousseau syndrome in different types of NSCLC has not been well established in the medical literature. Most published data and case series focus on lung adenocarcinoma, which appears to be the predominant NSCLC subtype associated with Trousseau syndrome.

A systematic review and meta-analysis found that the overall pooled incidence of thromboembolic events in patients with driver genes was 23% (95% CI 18–29) [2]. NSCLC patients with ALK rearrangements have the highest incidence of VTE, at 37% (95% CI 23–52), ROS1 rearrangements also carry a high risk, with reported incidence rates for venous thromboembolism between 27% and 46.6% [3]. These thrombotic events characteristically cluster around the peridiagnostic period, with up to 48% occurring at or near the time of initial cancer diagnosis [3]. In contrast, patients with EGFR or KRAS mutations do not show a significantly increased risk for thromboembolism compared to wild-type, and incidence rates are not specifically elevated above baseline NSCLC rates [2]. Few studies have reported the incidence of ATE among ALK + or ROS1 + NSCLC. One study reported that among ROS1 + patients who experienced a thromboembolic event, 42.4% had an arterial event as their first thromboembolic event [8].

The underlying mechanisms of increased thromboembolism in ALK and ROS1 rearrangement NSCLC remain incompletely understood, but current evidence suggests that the hypercoagulability is linked to tissue factor overexpression [3]. Another study notes that the increased VTE predisposition is thought to be mediated in large part by high levels of extracellular mucin, which activates platelets via P-selectin/L-selectin interactions to promote thrombosis [9]. Specific clinical features have been shown to amplify this prothrombotic state. Previous multivariable analysis revealed that the presence of lymph node metastasis increases the odds of thromboembolic events, which is particularly prevalent in ROS1 rearrangement tumors [8].

Current data suggests that there is no evidence that ROS1 tyrosine kinase inhibitor therapy prevents thromboembolic complications. A multicenter study found that thromboembolic events occur throughout the entire disease trajectory in ROS1-positive NSCLC, regardless of whether patients receive chemotherapy or targeted therapy [10]. Furthermore, clinical cohort analyses reveal that the occurrence of peridiagnostic thromboembolic events does not significantly impair overall survival or objective response rates (ORR) to first-line TKIs in patients with ROS1 rearrangement NSCLC [8,10]. This suggests that while TKIs effectively shrink tumor burden, the underlying prothrombotic cascade may persist independently.

Both low-molecular-weight heparin (LMWH) and direct oral anticoagulant (DOAC) are recommended as the treatment of choice for cancer-associated venous thrombosis in Trousseau syndrome [11]. For cancer-associated arterial stroke, no high-quality evidence establishes that anticoagulation is superior to antiplatelet therapy. Unlike cancer-associated VTE, where multiple randomized controlled trials support DOAC or LMWH use, secondary stroke prevention in cancer-associated cerebral ischemia lacks a comparably robust evidence base. The use of anticoagulation in cancer-associated arterial embolism has largely relied on extrapolations from venous thromboembolism trials, and this is a limitation widely acknowledged in the literature. Therefore, individualized, multidisciplinary decision-making between neurology and oncology is essential before initiating anticoagulation therapy in this patient population. LMWH has been preferred over vitamin K antagonists for this indication, but it requires daily injections and carries higher bleeding risk, including intracranial hemorrhage, which is a particular concern after ischemic stroke. DOACs are an emerging alternative, but randomized data are still limited [6]. There are currently no specific guidelines recommending routine thromboprophylaxis for ROS1 rearrangement NSCLC. In our case, DOAC was chosen over LMWH, taking into account her large ischemic stroke and the risk of intracranial hemorrhage.

DOACs and crizotinib have a clinically significant drug–drug interaction mediated by crizotinib’s inhibition of CYP3A4 pathways [12]. All DOACs are substrates of P-glycoprotein, and apixaban and rivaroxaban also undergo substantial CYP3A4 metabolism. Crizotinib is classified as a moderate CYP3A4 inhibitor without significant P-glycoprotein inhibition, placing it in a category where the combination carries limited clinical data but a plausible increase in apixaban exposure and bleeding risk. Guidelines remain inconsistent regarding crizotinib’s classification of CYP3A4 and P-glycoprotein inhibitory potency and whether it necessitates a mandatory apixaban dose adjustment or avoidance. The Anticoagulation Forum does not recommend a specific dose reduction. Instead, it advises clinicians to consider bleeding risk mitigation strategies [13]. In contrast, the European Heart Rhythm Association and the European Respiratory Society advise against the concurrent use of crizotinib with DOACs altogether [14].

Managing recurrent stroke in this setting presents a delicate balance between controlling active tumor-mediated hypercoagulability and mitigating the risk of hemorrhagic transformation when switching between antiplatelet agents and anticoagulants. Cancer patients have both a higher baseline risk of recurrent VTE and a higher risk of major bleeding on anticoagulation than non-cancer patients [15]. This poses a difficult treatment dilemma, balancing the competing risks of thrombosis and bleeding after initiating anticoagulation. In patients presenting with acute arterial ischemic stroke, primary or secondary anticoagulation must be carefully weighed against the formidable risk of hemorrhagic transformation and fatal subarachnoid hemorrhage, as exemplified in our case. Lung cancer is not among the gastrointestinal, urothelial, or gynecologic cancers associated with the highest DOAC-related bleeding risk. Apixaban, rivaroxaban, or edoxaban are all generally acceptable first-line options. LMWH remains preferred when there is impaired renal function, thrombocytopenia, malabsorption or low body weight [16].

The diagnosis of Trousseau syndrome often has no single validated diagnostic test or formal criteria. The syndrome is often diagnosed by exclusion. This poses significant clinical challenges and is particularly relevant when Trousseau syndrome presents as arterial or cerebrovascular thrombosis, since this presentation is less common than venous thromboembolism, carries high mortality, and can precede the cancer diagnosis entirely, leading to delayed or missed recognition [17].

Cancer patients who are presented with Trousseau syndrome have a higher mortality rate compared to cancer patients without this syndrome. Despite aggressive anticoagulation and antitumor treatment management, outcomes remained poor [1]. In our case, the patient had recurrent episodes of ischemic stroke despite the use of apixaban. Previous literature has also reported Trousseau syndrome patients who suffered recurrent thrombotic events while under anticoagulation therapy [18]. This highlights that thrombotic events may persist even with appropriate anticoagulant treatment, often leading to a poor prognosis. It emphasizes the importance of proactive monitoring and tailored management strategies for these affected patients.

Several limitations of this report warrant consideration. First, as a single case report, our observations lack generalizability regarding optimal anticoagulation in ROS1 rearrangement arterial thromboembolism. Second, transesophageal echocardiography was deferred due to critical illness, leaving occult non-bacterial thrombotic endocarditis or cardiac micro-emboli not fully excluded. Third, specialized thrombophilia panels (such as antiphospholipid antibodies or protein C/S deficiency) were not performed. Finally, the absence of a post-mortem autopsy precluded histological verification of microvascular thrombosis.

Trousseau syndrome is a recognized complication in patients with lung cancer. This case highlights that thromboembolic events can significantly compromise clinical outcomes, necessitating heightened clinical suspicion and early intervention.

## Figures and Tables

**Table 1:** Timeline of Clinical Course, Diagnostic Findings, and Management.

Time	Clinical Events
23 days before admission	Pathology proven adenocarcinoma of lung
19 days before admission	Brain MRI revealed no brain metastasis or intracranial lesions.
Day 1 of admission	Admission to ward for acute altered mental status. Brain MRI showed acute bilateral embolic infarctions. Initiated aspirin.
Day 2	FISH confirmed ROS1 rearrangement. Initiated crizotinib.
Day 5/ICU Day 1	Acute respiratory failure secondary to pneumonia. Intubated, transferred to ICU
Day 6/ICU Day 2	New right-sided hemiparesis. Brain CTA confirmed acute left M1-MCA occlusion.
Day 13/ICU Day 9	Switched aspirin to apixaban
Day 19/ICU Day 15	Recurrent neurological decline with left-sided weakness. Brain CTA showed right distal M1-MCA occlusion, left MCA hemorrhagic transformation, and right SAH. Discontinued apixaban. Neurocritical supportive care.
Day 26/ICU Day 22	Deep coma, refractory hypotension, severe bradycardia. CPR performed for 30 min without ROSC and pronounced deceased.

## Data Availability

All data generated or analyzed during this study are included in this published article.

## Abbreviations

The following abbreviations are used in this manuscript:

ALK	Anaplastic lymphoma kinase
aPTT	Activated partial thromboplastin time
ATE	Arterial thromboembolic events
CT	Computed tomography
CTA	Computed tomography angiography
DIC	Disseminated intravascular coagulation
EGFR	Epidermal growth factor receptor
FDP	Fibrin degradation products
FISH	Fluorescence in situ hybridization
ICU	Intensive care unit
INR	International normalized ratio
LMWH	Low molecular weight heparin
MCA	Middle cerebral artery
MRI	Magnetic resonance imaging
NOACs	Novel oral anticoagulants
NSCLC	Non-small-cell lung cancer
PD-L1	Programmed death-ligand 1
PT	Prothrombin time
ROS1	Proto-oncogene tyrosine-protein kinase 1
SAH	Subarachnoid hemorrhage
VTE	Venous thromboembolism
KRAS	Kirsten rat sarcoma
